# Improvement of Interaction in a Composite Structure by Using a Sol-Gel Functional Coating on Carbon Fibers

**DOI:** 10.3390/ma10090990

**Published:** 2017-08-25

**Authors:** Anna Szczurek, Michał Barcikowski, Karol Leluk, Bartosz Babiarczuk, Jerzy Kaleta, Justyna Krzak

**Affiliations:** 1 Department of Mechanics, Materials Science and Engineering, Faculty of Mechanical Engineering, Wroclaw University of Science and Technology, 25 Smoluchowskiego, 50-370 Wroclaw, Poland; anna.szczurek@pwr.edu.pl (A.S.); michal.barcikowski@pwr.edu.pl (M.B.); bartosz.babiarczuk@pwr.edu.pl (B.B.); jerzy.kaleta@pwr.edu.pl (J.K.); 2 Department of Environmental Engineering, Wroclaw University of Science and Technology, 9 Grunwaldzki Square, 50-377 Wroclaw, Poland; karol.leluk@pwr.edu.pl

**Keywords:** thin layer, silica, composite, adhesion, wettability

## Abstract

The modification of carbon fibers for improving adhesion between fibers and an epoxy resin in composite materials has become the focus of attention. In this work the carbon fiber coating process has been devised in a way preventing the stiffening and clumping of fibers. To improve interactions between coated fibers and a resin in composites, four types of silica coatings with different organic functional groups (3-aminopropyl–coating 1, 3-mercaptopropyl–coating 2, 2-(3,4-epoxycyclohexyl) ethyl–coating 3, methyl–coating 4) were obtained. Scanning electron microscopy (SEM) and atomic force microscopy (AFM) were used to distinguish the changes of a carbon fibers surface after coating deposition. The thickness of the obtained coatings, including the diversity of thickness, was determined by transmission electron microscopy (TEM). The increase in surface free energy (SFE) of modified fibers, including the distinction between the polar and dispersive parts, was examined by wettability measurements using a tensometric test. The developed coating preparation process allowed to cover fibers separately with nanoscale silica layers, which changed their morphology. The introduction of organic functional groups resulted in surface free energy changes, especially an increase in specific polar surface energy components.

## 1. Introduction

Nowadays the weight of construction materials plays a crucial role in industry, especially in automotive and aerospace industry, where a decrease in mass significantly reduces the cost of use. Commonly-used construction materials, due to their good mechanical properties and low weight, are carbon fiber reinforced plastics (CFRP). Despite many advantages of such materials, they are also characterized by structure defects, partly caused by the insufficient adhesion of reinforcing fibers to a matrix [1,2,3]. The defects of fiber matrix adhesion significantly affect the tensile and flexural strength of a composite material, promote crack growth leading to decreased toughness, fatigue strength, and impact resistance, they also facilitate the infiltration of liquids from the environment, which leads to corrosion.

The adequate adhesive strength of a composite is the key to the determination of mechanical performance [2]. Adhesion is provided by physical and chemical interactions between two phases [4]. In the case of CFRP these two phases are carbon fibers and a polymer, e.g., epoxy resin. The physical interactions depend on the surface properties of fibers, such as morphology and topography, and also on the affinity between functional groups of composite components. The chemical interactions, which are very strong, occur when chemical bonds are created, e.g., between reactive functional groups of the fiber surface and the resin.

The crucial role in the creation of both types of interactions is played by functional groups. However, carbon fibers, being composed of carbon, except for very few functional groups which can arise through the oxidation of carbon, are chemically inert, which makes the formation of interfacial strength difficult. Carbon fibers, being composed of carbon, exhibit non-polar characteristics. They do, however, contain an amount of oxygen bonded with carbon in the form of carbonyl, oxide, hydroxyl, and even carboxyl functional groups. These groups are beneficial for the wettability of carbon fibers by epoxy resins and their hardeners, as well as for the emergence of interphase adhesive bonds. The number of these groups is, however, insufficient, so carbon fibers are subjected to surface treatment [5]. There are many methods of improving their adhesion properties, e.g., plasma treatment [6], coronal discharge [5], heat treatment [3], electrochemical treatment [7], oxidizing with nitrous acid (HNO_3_) [5], coatings deposition [2,8]. The adhesion between the coating and carbon fibers is very important for the cohesion of composite products, but due to the small thickness of coating (in the case described in this paper less than 50 nm) direct measurements of its adhesion to the fiber are difficult mainly because of the measurement resolution of the equipment. Typically, such measurements are carried out on samples of the target composite material (i.e., fibers + coating + matrix). However, this stage of research was not the purpose of this publication. 

**Figure 1:**
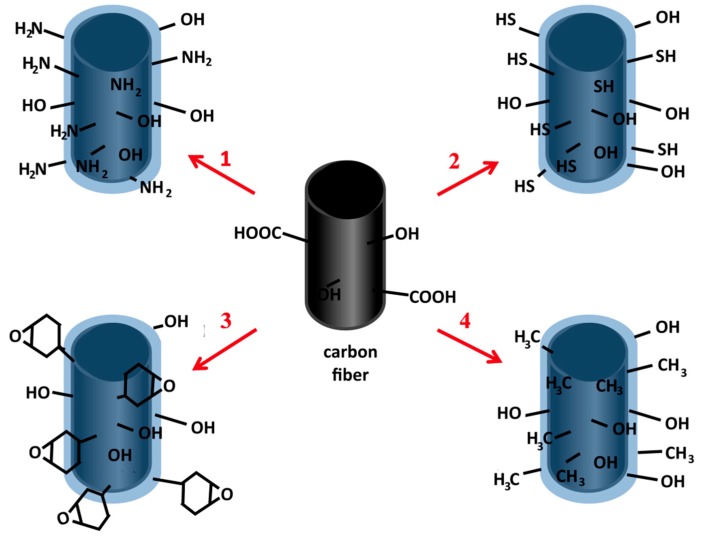
Scheme of carbon fiber surface functionalization by the sol-gel coatings (with numbers compatible with numbers of coatings described in manuscript).

One type of coatings that can be used as pro-adhesive coatings on carbon fibers are oxide coatings obtained by the sol-gel method [9,10,11], the method which nowadays substantially contributes to the creation of highly-advanced nanostructured materials with required, very specific, properties, e.g., hybrid, templated, with controlled porosity [12,13], not only in the form of layers [14,15]. Moreover, the sol-gel method is considered eco-friendly, because of the synthesis process, especially in comparison to traditional methods of obtaining glass and ceramics and can still become “greener” [16]. By utilizing phenomena in the liquid phase this method allows the use of a relatively simple method of coating deposition. Additionally, the sol-gel synthesis based on hydrolysis and condensation reactions allows to obtain organically functionalized oxide coatings through the diversity of precursors which can be used during synthesis. The use of a precursor which has an non-hydrolysable organic group in their structure allows to obtain a layer with this group as a functional group in the oxide network, which can interact and/or bond with groups of other materials, with which the interaction (also adhesion) should be improved. Moreover, these interactions can also be modified by other properties of sol-gel materials, such as overall charge, number of silanol groups, which can be determined during synthesis [17]. The idea of the creation of an organically functionalized silica coating is shown in Figure 1. The presence of appropriate groups on the surface of fibers should be strongest determinants of the increase in the interaction with the matrix.

In this work, the effect of carbon fiber modification by organically functionalized sol-gel coating deposition is presented. Changes in the topography and morphology of carbon fibers after coating deposition are examined with microscopic studies. In addition, the IR investigations approximate the structure of the obtained coatings. The effect of the surface modification influence on the ability to create interaction is also studied by the wettability test and the surface free energy values for coated and uncoated fibers are determined. We expected to observe the changes of the fibers’ surface properties after modification and differences in coating morphology, topography, and wettability, depending on the functional groups present in individual silica coatings.

## 2. Results

### 2.1. SEM

**Figure 2:**
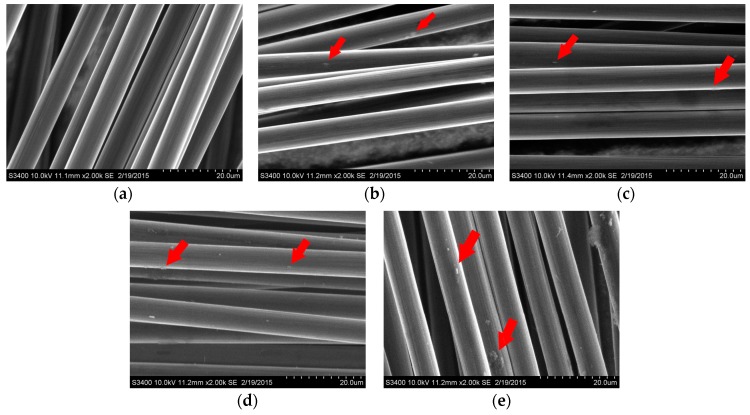
SEM micrographs of uncoated carbon fibers (**a**) and carbon fibers modified with coating 1 (**b**); coating 2 (**c**); coating 3 (**d**); and coating 4 (**e**).

The SEM micrographs of modified and unmodified carbon fibers with magnification 2000×, as the most informative ones in the determination of the uniformity of the structure or the lack of formation of cracks, chipping or other defects characteristic of sol-gel layers [18,19], and in the preclusion fiber bonding, are presented in Figure 2. The surface morphology of coated and uncoated fibers is not significantly different, however, some local and very small accumulations of layer material (indicated with an arrow) are observed, the most in the case of coating 4. According to visual observations, which show the lack of fiber bonding and stiffening, SEM investigations indicate that fibers are separated and were covered individually.

### 2.2. AFM

**Figure 3:**
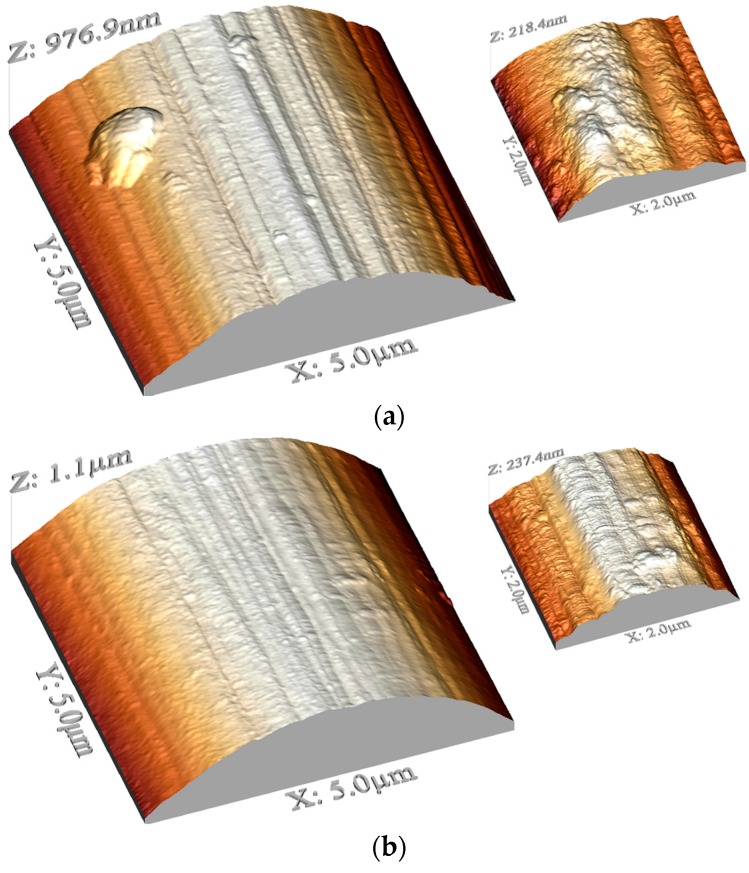

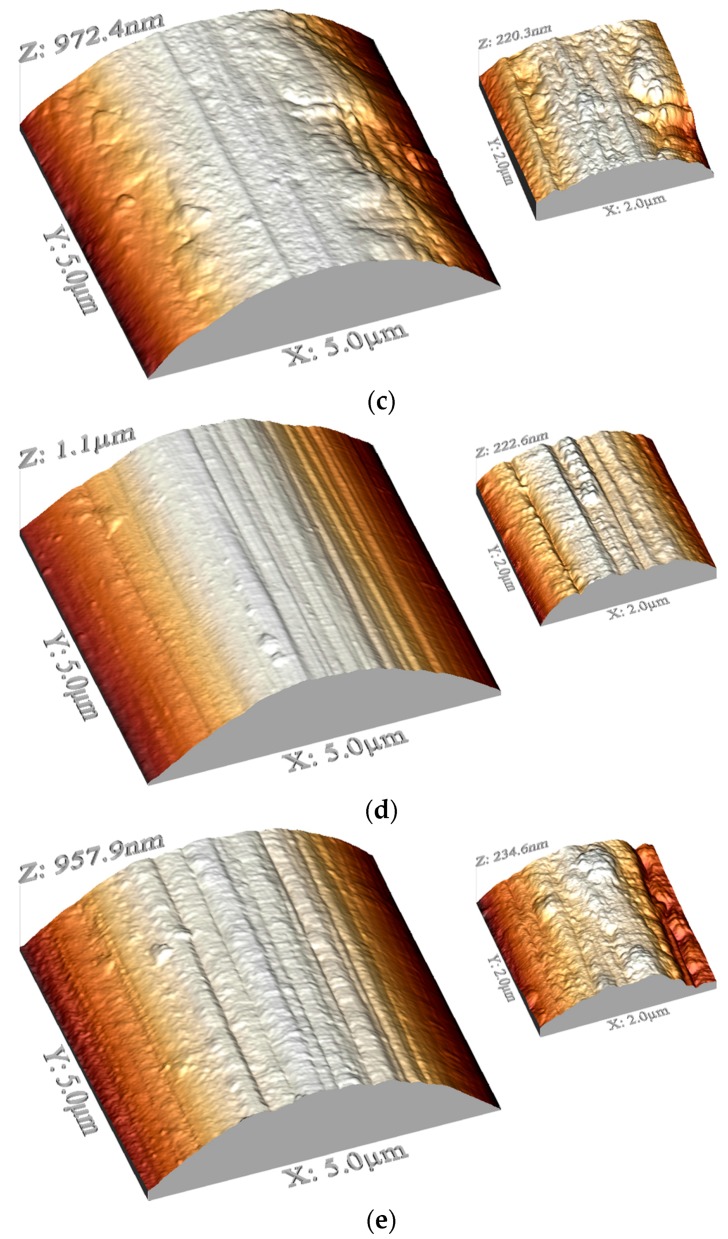
AFM images of uncoated and coated carbon fibers in 3D mode: carbon fibers without coating (**a**); and carbon fibers modified with coating 1 (**b**); coating 2 (**c**); coating 3 (**d**); and coating 4 (**e**).

**Figure 4:**
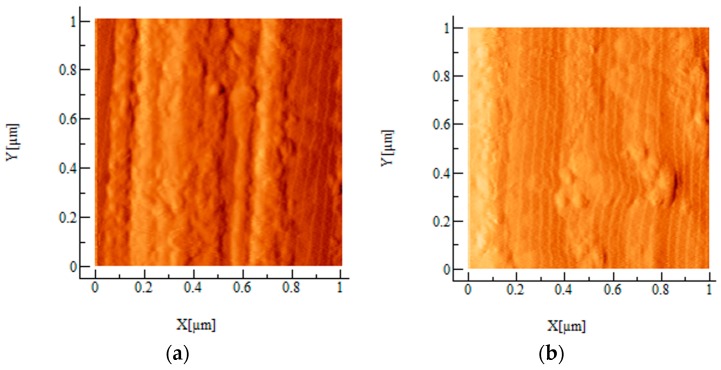

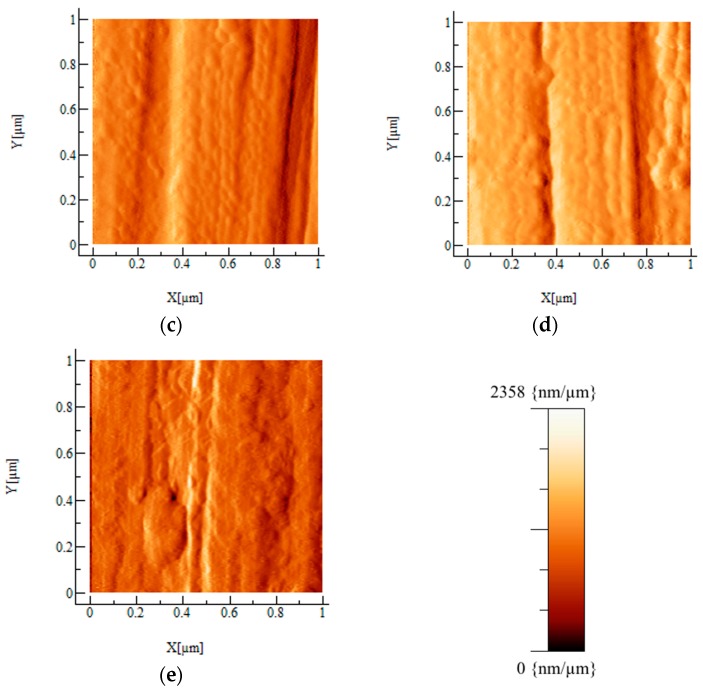
AFM images of uncoated and coated carbon fibers in 2D derivative mode: carbon fibers without coating (**a**); and carbon fibers modified with coating 1 (**b**); coating 2 (**c**); coating 3 (**d**); and coating 4 (**e**).

AFM examinations revealed changes in the topography of carbon fibers after coating application. The topographic 3-D profiles of fibers in the area 5 μm × 5 μm and 2 μm × 2 μm are presented in Figure 3. All fibers reveal the presence of grooves along the length of fibers, which is characteristic of carbon fibers made by carbonizing a precursor material (usually poly (acryl nitrile)). In the case of fibers coated with coating 2, grooves are the least distinct, which can be caused by the texture of this coating. The roughness of fibers with coating 1 and 3 is similar to uncoated fibers, in other cases fibers after modification are apparently rougher. Greater roughness is manifested by a higher surface area, so also by more potential sites for interactions, which should result in improved adhesion. To show the topography in higher magnification, 2-D images in a derivative mode in the area 1 μm × 1 μm are presented in Figure 4.

### 2.3. TEM and EDS

**Figure 5:**
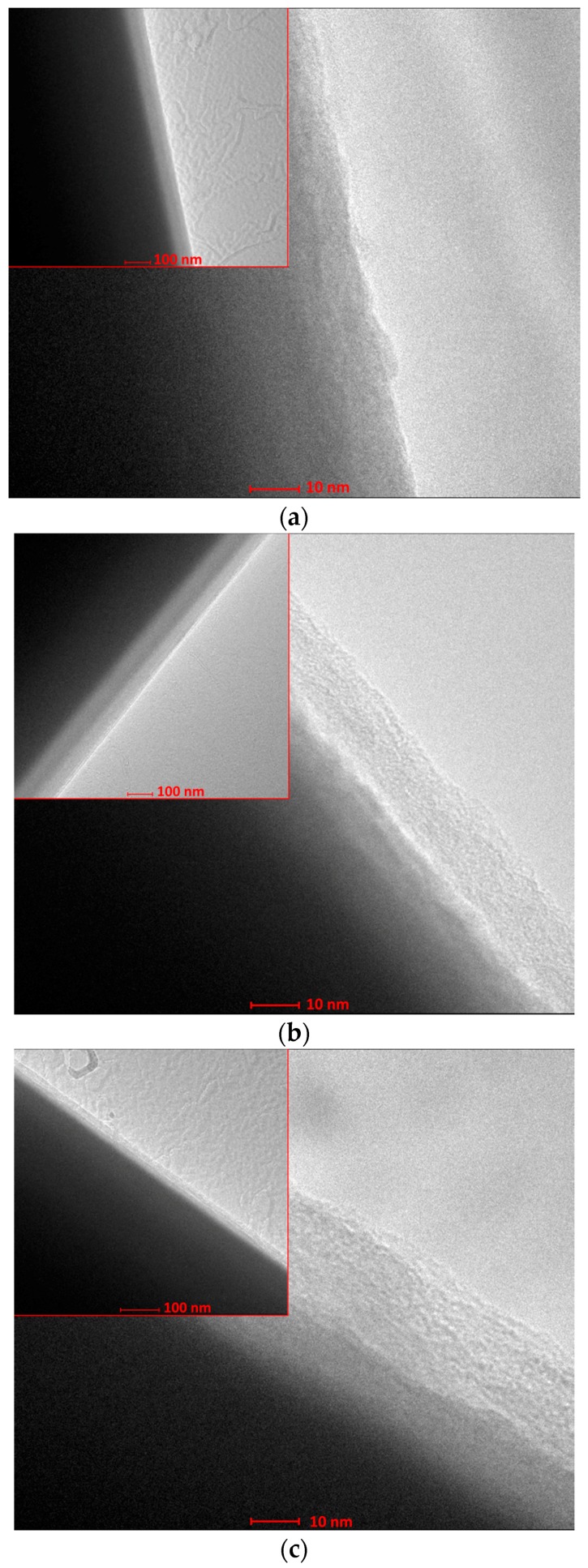

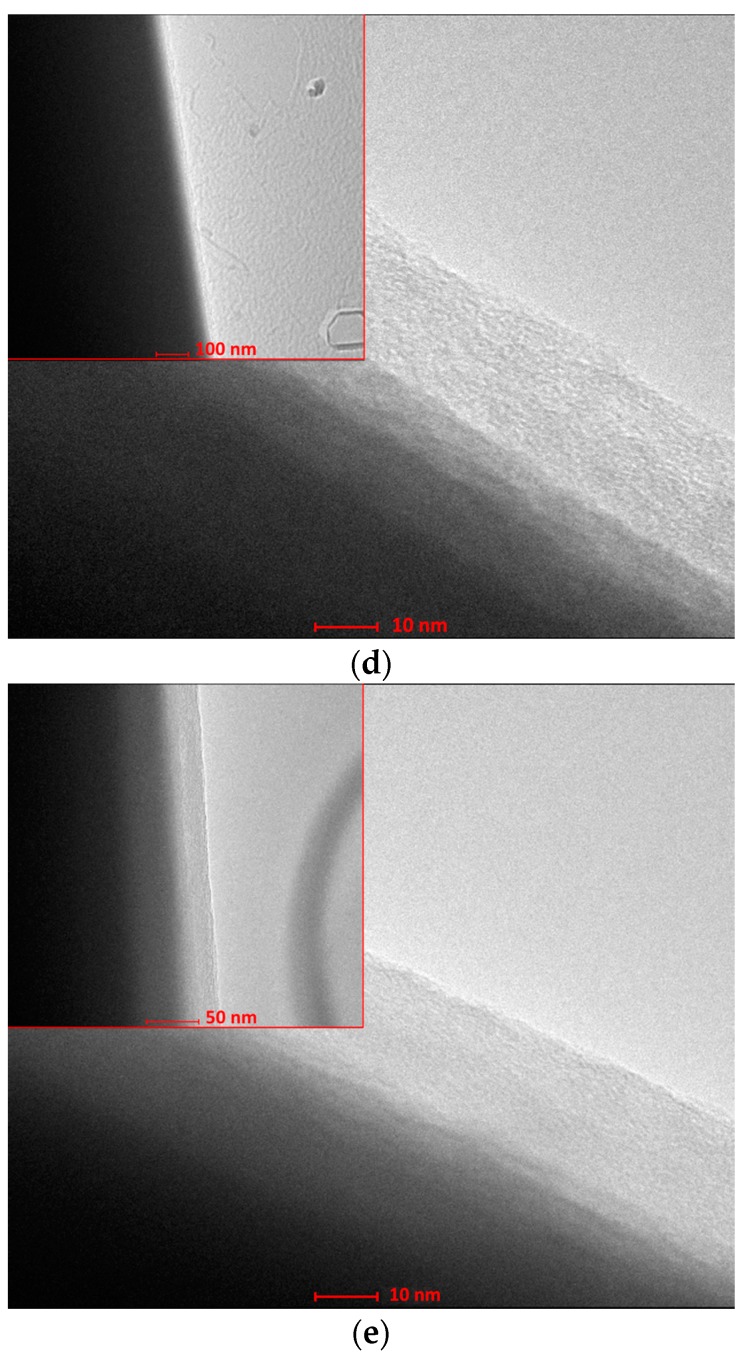
TEM images of uncoated carbon fibers (**a**) and carbon fibers modified with coating 1 (**b**) coating 2 (**c**); coating 3 (**d**); and coating 4 (**e**).

**Figure 6:**
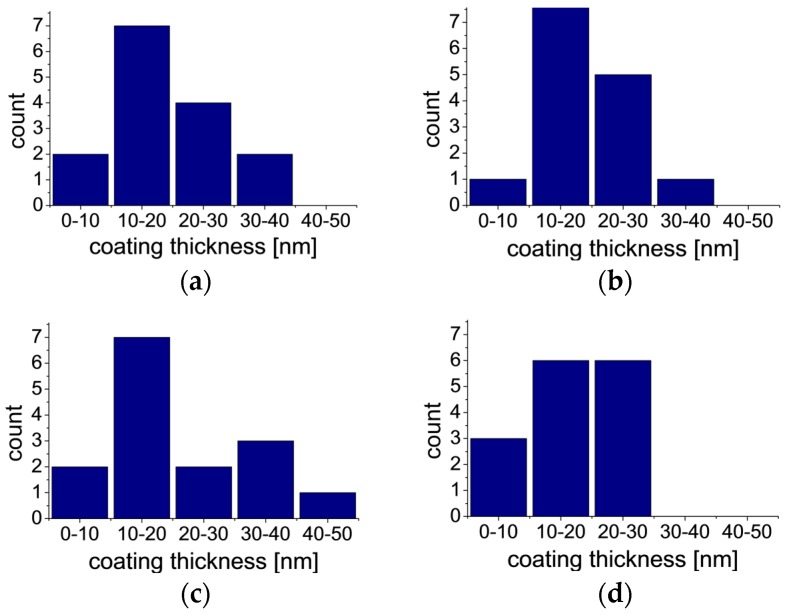
Histograms of measured thickness of coating 1(**a**); coating 2 (**b**); coating 3 (**c**); and coating 4 (**d**).

Figure 5 presents TEM micrographs of uncoated and coated fibers. The pictures show that coatings were successfully applied. The obtained coatings were continuous but they varied in thickness. In most cases the thickness of coatings was close to 20 nm, however, some deviations, presented on histograms (Figure 6), were observed. The histograms of thickness for each type of coatings, presented in Figure 6, illustrate the diversification of layer thickness. The thickness of coating 3 is the most diverse and only for this material the coating thickness reaches a value higher than 40 nm. The smallest thickness variations are observed for coating 4. The low thickness of the obtained layers determines the retention of the fiber flexibility.

**Figure 7:**
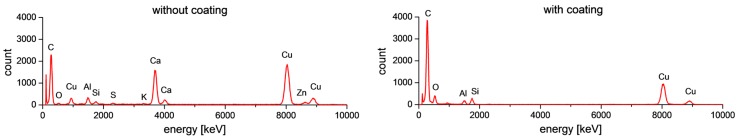
EDS results for carbon fibers without coating and with coating 2.

The elemental composition of layers was determined by the EDS analysis, which affirms that the resulting coatings are silica coatings. All EDS graphs of coated carbon were similar—the example is presented in Figure 7. Small peaks from Si atoms were observed on the graphs. The small amount of silica results from the small thickness of coatings. In the case of coated carbon fibers, aside from the silica, only carbon, oxygen, and elements coming from the background (copper and aluminum) are detected. In addition, the amount of C, relative to the uncoated fiber, increased indicating the presence of the organic moieties of the applied coating. In the case of uncoated carbon fibers, elements which indicate some remaining contaminations or some residual sizing on carbon fibers are observed, however, they were finally removed after the coatings deposition process.

### 2.4. IR

**Figure 8:**
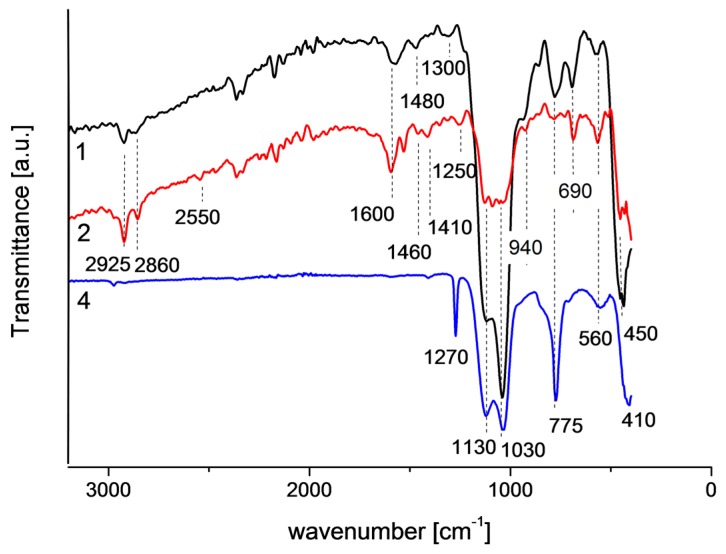
FTIR spectra of the powder obtained from sols used to coating preparation (1: powder from sol used to prepare coating 1; 2: powder from sol used to prepare coating 2; and 4: powder from sol used to prepare coating 4).

FTIR spectra of powders corresponding to individual sols are presented in Figure 8. In each of the spectra a strong band in the range of 1000–1200 cm^−1^, characteristic for asymmetric stretching of Si-O-Si [20], with two maxima at 1030 and 1130 cm^−1^ is observed. The band at 1130 cm^−1^ is assigned to the Si-O stretching [21], the band at 1030 cm^−1^ to Si-O-Si skeletal vibrations which are characteristic for poorly cross-linked acid catalyzed gels [22]. The weak band at about 1250 cm^−1^ is assigned to antisymmetric Si-O-Si stretching longitudinal optical mode -LO_3_ [20,23]. The band characteristic for the rocking Si-O-Si vibration [20] is observed in spectra 1 and 2 at about 450 cm^−1^, in the case of spectrum 4 this band is probably overlapped by the band at 410 cm^−1^. Weak bands at 560 cm^−1^ are assigned to symmetric Si-O-Si stretching [24]. Moreover, in all spectra, the band identified as the bending Si-O-Si vibration, in which oxygen moves approximately at right angles to the Si–Si lines and in the Si-O-Si planes [20], is observed at 775 cm^−1^. The weak band characteristic for symmetric stretching of Si-OH [20] is observed at 940 cm^−1^ in spectra 1 and 2. The band at 690 cm^−1^ band is related to the Si-O or plane Si-O^−^ vibrations [25].

In the case of spectrum 4, where methyltrimethoxysilane was used as organically functionalized precursor, a sharp band is observed at 1270 cm^−1^. This band is characteristic for methyl groups attached to silicon, due to the symmetric deformation vibration of the CH_3_ group [24]. The weak band at 2550 cm^−1^ in spectrum 2 is associated with the S-H stretching and deformation vibration of the mercapto group [24], which is present in the organically functionalized precursor used to obtain sol 2. Moreover, the bands at 1410 and 1460 cm^−1^, associated with the deformation vibrations of CH_2_ in -CH_2_-S- groups [24], are observed. The band observed at 1480 cm^−1^ in spectrum 1 is assigned to the NH_2_ deformation modes of the amine groups, very strongly hydrogen bonded to the silanol groups [26]. The bands at 2860 and 2925 cm^−1^ are attributed to symmetric and antisymmetric C-H stretching vibrations of CH_2_ groups [24], respectively. The bands which appeared at 1600 cm^−1^ are associated with O-H stretching vibrations of water [24].

### 2.5. Wettability

**Figure 9:**
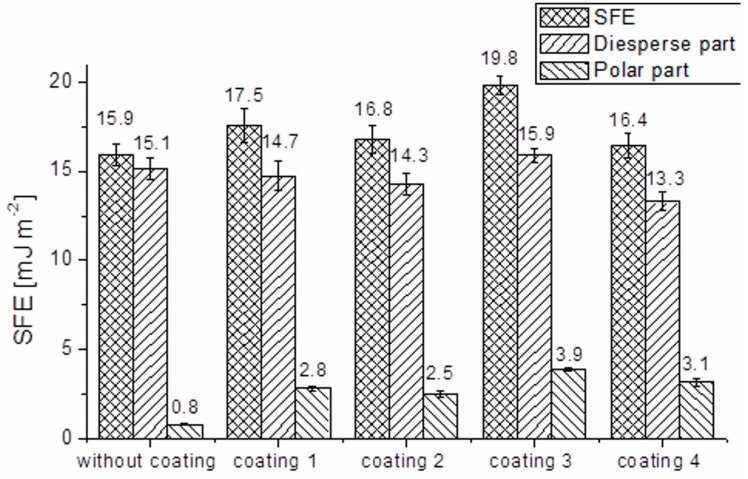
Surface free energy of fibers.

The results obtained for the surface free energy (SFE) with distinguished dispersive and polar components are presented in Figure 9. The SFE for uncoated fibers is equal to 15.9 mJ·m^−2^, of which the dispersive component is 15.1 and the polar component is 0.8 mJ·m^−2^ indicating the polar character at the level of 5.1%. The total free energy significantly increased in the case of fibers with coating 3 and reached 19.8 mJ·m^−2^. In the other cases only a slight increase in the total free energy is observed but for all coated fibers the polar component is substantially higher than for the uncoated ones–an increase in the value from 0.8 to 2.5–3.9 (depending on the layer). Thus, as a result, the polar character as the percentage of polarity, presented in Table 1, for the processed samples reveals a substantial increase in values which is 3–4 times higher comparing to the uncoated fibers. The increase in the polar component of the surface free energy is caused by the introduction of polar groups on the surface of the applied coatings–the organic functional groups of precursors and/or -Si-OH groups. It can be concluded that the interactions between the coated carbon fibers and the matrix will depend on the polarity of functional groups. However, it is striking that the polar component of the surface free energy is higher for the coating with a methyl group (H_3_C-)–coating 4, than with 3-mercaptopropyl group (HS(CH_2_)_3_-)–coating 2. It can be caused by the inductive effect of alkyl groups–electrons can move toward hydroxyl groups (HO-), which also occurs in the silica coating structure, thereby increasing the electron density of these groups.

## 3. Discussion

Carbon fiber modification was performed by the application of organically-functionalized silica sol-gel coatings to improve their physical and chemical interaction with the matrix (epoxy resin) in composite materials. The literature data indicate that modification or even functionalization (which was the case here) may influence interaction on the fiber/matrix border [27,28,29]. The changes in surface morphology, topography, and wettability after the modification were analyzed in this study. The authors developed the parameters of the sol synthesis and coating application process, which allowed retaining the flexibility of carbon fibers and avoid fiber clumping. These aspects are crucial for the use of modified fibers for composite preparation and the method of isolating the fibers when the coating process is not obvious in the area of fiber surface modification [30,31]. The surface topography measurements showed that the deposition of a coating with mercapto functional groups (-SH) and also with methyl functional groups (-CH_3_) increases the roughness of fibers. In the other two cases no significant changes in surface texture were observed. The increased roughness is associated with bigger specific surface area, which results in more active sites on the fiber surface for better mechanical interlocking of the matrix with fibers, and this is consistent with the data presented by Tiwari et al. [32]. The investigations by TEM and EDS revealed the presence of continuous silica coatings about 20 nm thick. The variations of thickness may result from the complexity of the structure of the non-hydrolysable functional group (H_2_N(CH_2_)_3_- for coating 1, HS(CH_2_)_3_- for coating 2, CHOHC_4_H_8_-(CH_2_)_2_- for coating 3 or H_3_C- for coating 4). The highest thickness values and the largest dispersion of these values are observed for coating 3 which contains, sterically, the largest non-hydrolysable functional group. This small thickness of coatings and the presence of organic moieties, which constitute the coatings, in the silica network determine the maintenance of fiber flexibility. IR studies of powders obtained from sols used in coatings preparation confirm the formation of organically-modified silica networks, such as expected. The increase in the polar part of SFE, which is observed for all coatings, should facilitate the formation of interaction with the matrix in composites. In this investigation, a significantly stronger influence of the functional group than the impact of roughness on the surface free energy is observed. In the case of the coating with epoxy groups the improvement of interaction strength will be the biggest due to the increase in the total surface free energy of fibers from 15.9 to 19.8 mJ·m^−2^. In the case of this coating, the range of SFE changes is similar to other sol-gel coatings on carbon fibers recently described in the literature [33]. This value has a direct impact on the adhesion [2]. The components of the surface free energy are physically associated with the work of adhesion *W_ad_*. The work of adhesion can be estimated by applying the Young-Dupré equation and the Fowkes theory according to the Equation (1) [2]:

(1) Wad=2(γfDγrD)12+2(γfSPγrSP)1/2

where *W_ad_*: work of adhesion, $\gamma_{f}^{D}$: dispersive surface energy components of the fibers, $\gamma_{r}^{D}$: dispersive surface energy components of the resin, $\gamma_{f}^{SP}$: specific polar surface energy components of the fibers, and $\gamma_{r}^{SP}$: specific polar surface energy components of the resin.

Substituting the literature value of SFE of cured epoxy resin ($\gamma_{r}^{D}\;$= 37.71 mJ·m^−2^, $\gamma_{r}^{SP}\;$= 8.07 mJ·m^−2^) [3] in the above Equation (1) with the work of adhesion for the best obtained coating (coating 3) is determined as 62 mJ·m^−2^. It represents a growth of 16% in comparison to the work of adhesion for uncoated carbon fibers equal to 53 mJ·m^−2^.

The subject of the adhesion improvement of carbon fibers and resin is still interesting and needs to be developed. In the previously published research different types of pro-adhesion coatings are described and similar aspects of fibers modifications are discussed [27,28,29]. Despite this, it is desirable to increase the roughness of carbon fibers, the aim is not always achieved in research, in some cases the smoothing effect is observed [34]. The thickness of coatings obtained by different researchers is expressed at the nanoscale, but both with thickness of several dozen nm [35] (as in this work) and also of several hundred nanometers [10]. In the case of sol-gel coatings on carbon fibers, the problem with defects and cracks appearing with the increasing thickness of coatings was observed by Li et al. [9]. The wettability changes in similar research show a comparable trend–the increase in SFE is mainly based on the increase in the polar component [27,34,36]. The materials presented in this work were obtained with the exclusion of the defects described in the literature, such as fiber clumping [30,31], and also coating spalling and crushing [9,31].

The obtained results indicate a potential for the application of the received coatings as the pro-adhesive coatings in composite materials. Synthesized sol-gel coatings suggested a good influence on the strength adhesion of CFRP.

## 4. Materials and Methods

### 4.1. Materials Preparation

Carbon fibers (Toho Tenax Co., Ltd., 5631 12 k, Tokyo, Japan) were cleaned in an ultrasonic bath (in three steps: with acetone, with water and with ethanol) and coated by four types of coatings. Cleaned and uncoated fibers were used as a comparative sample. All sols used for coating deposition were obtained by the hydrolysis and condensation reactions of a mixture of two precursors: tetramethoxysilane, TMOS (Sigma Aldrich, St. Louis, MO, USA), and an organically-functionalized precursor, (3-aminopropyl) trimethoxysilane–APtMOS (Sigma Aldrich) in sol 1, (3-mercaptopropyl) trimethoxysilane–MercPtMOS (Alfa Aesar, Haverhill, MA, USA) in sol 2, 2-(3,4-epoxycyclohexyl) ethyltrimethoxysilane–EpoxcHtMOS (abcr, Karlsruhe, Germany) in sol 3, and methyltrimethoxysilane–MtMOS (Sigma Aldrich) in sol 4. In sol 1 the solvent was methanol–MeOH (POCH, Gliwice, Poland) and in sols 2, 3, 4 it was ethanol-EtOH (POCH). In each case reactions were catalyzed with hydrochloric acid–HCl (Stanlab, Lublin, Poland). Molar ratios of reagents for particular sols are shown in Table 2. With such fixed molar ratios, in each case, homogeneous stable hydrolysates were obtained by 1.5 h of stirring. Layers were deposited in an ultrasonic bath of fibers dipped in particular sols and left immersed overnight. The excess of coatings (without previous drying) was rinsed in methanol with the use of ultrasounds to obtain fibers which are elastic and not clumped together. Fibers with thinned coatings were removed from the methanol and dried in air. The deposited coatings were stabilized by temperature treatment for 12 h at 100 °C with the controlled ramp rate (1 °C·min^−1^). In order to perform structural studies by IR spectroscopy, portions of sols were dried and thermally treated in the same way as the layers and grinded in mortar to powder. (In the case of sol 3 it was impossible to receive the powder). This procedure was used due to the insufficient thickness of layers to perform spectroscopic studies on coated fibers.

### 4.2. SEM

The surface morphology of uncoated and coated carbon fibers was examined with a scanning electron microscope (SEM) S-3400 N (HITACHI, Ltd., Tokyo, Japan) at the Laboratory of Sol-Gel Materials and Nanotechnology, Wroclaw University of Science and Technology. Prior to SEM examinations the samples were sputtered with carbon. The investigations were carried out by an SE detector with the following magnifications: 100×, 250×, 500×, 1000×, 2000×, 3500×, and 10,000×.

### 4.3. AFM

The surface topography of uncoated and coated carbon fibers was measured with an atomic force microscope (AFM) dimension scanning probe microscope (Veeco-Bruker, Billerica, MA, USA) at the Advanced Materials Engineering and Modelling Group, Wroclaw University of Science and Technology. During the investigations the tapping mode was used. The following scan sizes were used: 5 µm × 5 µm, 2 µm × 2 µm, and 1 µm × 1 µm. The data were compiled using WSxM [37].

### 4.4. TEM and EDS

To confirm the presence of thin silica layers on the coated carbon fibers, examinations with a high-resolution transmission electron microscope (TEM) Tecnai G2 20 X-TWIN (FEI, Hillsboro, OR, USA), equipped with an EDS detector, were performed at the Laboratory of Electron Microscopy, University of Wroclaw. Based on the taken micrographs, the thickness of coatings was estimated. The thickness measurement was made at 15 areas for each type of coating.

### 4.5. IR

To approximate the structures obtained during coatings, the synthesis IR spectroscopy measurements of powders were carried out in the range of 400–4000 cm^−1^ using Vertex 70 v (Bruker Billerica, MA, USA) equipped with a diamond ATR cell at the Laboratory of Oscillatory Spectroscopy, Wroclaw University of Science and Technology. The spectra were measured with the resolution of 4 cm^−1^.

### 4.6. Wettability

Wettability tests were performed with a Sigma 700 (KSV Instruments, Helsinki, Finland), force tensiometer at the Department of Environmental Engineering, Wroclaw University of Science and Technology. The Washburn method was used to assess the wettability of fibers. In this method, a glass tube (holder) with a filter base filled with cut fibers comes into contact with a test liquid. The liquid is drawn up as a result of capillary action (cut fibers may be treated as a bundle of capillaries). The increase in the mass of the tube, which is suspended from a force sensor, is determined with respect to time during the measurement. The surface free energy (SFE), as well as its polar part and disperse part, were calculated from dynamic measurements. The polar and nonpolar liquid–water and diiodomethane–were used to determine wettability, whereas cyclohexane was used as the reference one. All chemicals were of analytical grade. For each individual sample five repetitions of measurements were performed.

## 5. Conclusions

Four different coatings—with different organic functional groups—have been obtained to change carbon fiber surface properties, which should translate into the improvement of interactions between fibers and epoxy resin and, finally, into better adhesion between these two components of construction composites. The coatings were obtained by the sol-gel method with the use of organically-functionalized silanes as precursors. The elaborated method of coating deposition allowed to receive the layers which do not stiffen the fibers and do not cause fibers to clump. After coating deposition the surface topography of fibers was changed by varying degrees depending on the layer composition. The thickness of the obtained coatings was differentiated, but did not exceed 50 nm. The spectroscopic studies of the obtained materials showed the developed oxide network structure and also confirmed the presence of organic functional groups. Thus, the key places for chemical interaction between fibers and epoxy resin matrix were provided. Wettability tests demonstrate that the increase in the SFE is the most significant in the case of the coating with epoxy functional groups, but in all coatings the increased participation of the polar component of the SFE was observed. Thus, by selection of the proper sol-gel pro-adhesive coatings for a chosen matrix, the formation of the interaction type and, thus, strengthening it, is achievable.

## Figures and Tables

**Table 1:** Polar character of fibers without and with individual coatings.

Sample	% Polarity
without coating	5.1
coating 1	16.1
coating 2	14.9
coating 3	19.7
coating 4	19.2

**Table 2:** Molar ratios of reagents for obtained sols.

Sol	Organically Functionalized Precursor	TMOS	Alcohol	HCl
1	1	0.3	10.6	0.1
2	1	0.3	7.9	0.1
3	1	0.5	12.1	0.1
4	1	0.2	6.1	0.1
